# A novel GIS-based ensemble technique for flood susceptibility mapping using evidential belief function and support vector machine: Brisbane, Australia

**DOI:** 10.7717/peerj.7653

**Published:** 2019-10-09

**Authors:** Mahyat Shafapour Tehrany, Lalit Kumar, Farzin Shabani

**Affiliations:** 1 School of Environmental and Rural Science, University of New England, Armidale, NSW, Australia; 2 Geospatial Science, School of Science, RMIT University, Melbourne, Australia; 3 ARC Centre of Excellence for Australian Biodiversity and Heritage, Global Ecology, College of Science and Engineering, Flinders University of South Australia, Adelaide, Australia; 4 Department of Biological Sciences, Macquarie University, Sydney, NSW, Australia

**Keywords:** Flood susceptibility mapping, Support vector machine, Evidential belief function, Ensemble modeling

## Abstract

In this study, we propose and test a novel ensemble method for improving the accuracy of each method in flood susceptibility mapping using evidential belief function (EBF) and support vector machine (SVM). The outcome of the proposed method was compared with the results of each method. The proposed method was implemented four times using different SVM kernels. Hence, the efficiency of each SVM kernel was also assessed. First, a bivariate statistical analysis using EBF was performed to assess the correlations among the classes of each flood conditioning factor with flooding. Subsequently, the outcome of the first stage was used in a multivariate statistical analysis performed by SVM. A highest prediction accuracy of 92.11% was achieved by an ensemble EBF-SVM—radial basis function method; the achieved accuracy was 7% and 3% higher than that offered by the individual EBF method and the individual SVM method, respectively. Among all the applied methods, both the individual EBF and SVM methods achieved the lowest accuracies. The reason for the improved accuracy offered by the ensemble methods is that by integrating the methods, a more detailed assessment of the flooding and conditioning factors can be performed, thereby increasing the accuracy of the final map.

## Introduction

Climate change and the inevitable urbanization have increased the occurrences of floods (Kjeldsen, 2010). The direct consequences of flooding include the loss of life, destruction of property, damage to crops, and deterioration of health conditions as a result of waterborne illnesses. Flooding can cause serious damages by dragging huge objects across the land on which the water flows (Fotovatikhah et al., 2018). Large floods can affect wildlife and decrease the level of biodiversity in inundated areas. A decrease in the habitat potential and food availability in the affected areas can cause long-term effects for the surviving wildlife. Population growth can result in increased constructions on floodplains. Smaller dwellings can be built that can result in denser cities and an increased possibility of floods in such areas. More closely constructed dwellings increases the quantity of houses that are potentially exposed to flood damage. Therefore, the costs involved in flood damage are considerably high in terms of both damaged assets and human fatalities. It is more important to aim at preventing such disasters than compensating for the damages. Preventive actions can minimize the possibly irreversible damages caused to buildings, farming, and transportation (Youssef, Pradhan & Sefry, 2016). The regions that are susceptible to floods must be identified to assist the governments and agencies in avoiding as much destruction as possible. It is not easy to determine the impact of a flood because it is not tangible; the evaluation requires a considerable amount of time. Conversely, the loss and destruction cause by a flood can be measured more easily (Yi, Lee & Shim, 2010).

There is a need for more studies based on floods and floodplain management strategies to improve the existing knowledge concerning the way floods occur under varying climate and catchment situations. Numerous studies have utilized flood susceptibility mapping (Merz, Thieken & Gocht, 2007; Pradhan, 2010; Pradhan & Youssef, 2011; Tehrany et al., 2014; Van Alphen et al., 2009). However, it remains to solve the problem of generating accurate flood forecasts and maps. The rainfall-runoff modeling techniques WetSpa, HYDROTEL, and SWAT are some of the popular hydrological methods (Herder, 2013). Calibration and sensitivity analysis must be performed for these methods (Neitsch et al., 2002). Moreover, it is not easy for researchers with limited expertise in hydrology to implement these methods (Herder, 2013). Therefore, they are less applicable for real-time studies. Bivariate statistical analysis (BSA) and multivariate statistical analysis (MSA) are two forms of quantitative (statistical) techniques (Ayalew & Yamagishi, 2005). BSA includes the analysis of the correlations between the flood inventory map and each conditioning factor (Althuwaynee, Pradhan & Lee, 2012). Each class of a particular conditioning factor is examined separately, and the final probability map is produced by the sum of all weighted summation. Frequency ratio (FR) and weight-of-evidence (WoE) are two examples of BSA methods. MSA methods such as logistic regression (LR) examine the multiple associations between the different conditioning factors and the flood inventory map simultaneously. LR evaluates the correlations between the different conditioning factors and flooding at the same time (Carrara, Crosta & Frattini, 2003). FR and LR have been widely used in studies based on natural hazards (Lee & Pradhan, 2007; Park et al., 2013); both the methods involve simple and linear calculation processes. On one hand, BSA neglects the correlations among the different conditioning factors, which is considered a disadvantage. On the other hand, MSA neglects the influence of the classes of each conditioning factor on the occurrence of floods (Tehrany, Pradhan & Jebur, 2013). Generally, catchments cannot be accurately modeled using simple and linear techniques owing to their complex, dynamic, and non-linear structure. Other available techniques include the WoE, evidential belief function (EBF), artificial neural network (ANN), support vector machine (SVM), and decision tree (DT) that are more advanced and structurally complex. The WoE method, which is a BSA method, is based on Bayesian theory, and it is appropriate for solving decision-making problems under uncertainties. This technique has been applied in several studies based on natural hazards (Pourghasemi et al., 2013b). However, a demerit of all BSA methods can also be observed in the WoE method: it does not evaluate the correlation among the different conditioning factors.

The SVM, ANN, and DT methods are known as machine learning methods. They can be trained using training datasets; then, the model can be applied to the whole dataset. Machine learning methods have been used in a variety of applications (Qasem et al., 2019). Fotovatikhah et al. (2018) have stated that the ANN method is one of the most popular computational intelligence (CI) methods; it was applied in flood mapping by Campolo, Soldati & Andreussi (2003), Shu & Burn (2004) and Seckin et al. (2013), among others. It can handle errors in the input dataset and gather information from incomplete or contradictory datasets. However, the accuracy of its outcomes decreases for cases in which the validation data has values beyond the range of those used to run the model (Kia et al., 2012). In cases where a large number of factors are used in the analysis, the entire modeling process becomes time consuming (Ghalkhani et al., 2013). An adaptive neuro-fuzzy inference system (ANFIS) (Dehghani et al., 2019) is an integrated method created using the ANN and fuzzy interface system (FIS) methods. This method has better capability than the individual ANN method (Tehrany, Pradhan & Jebur, 2014).

The application of the DT method in flood susceptibility analysis has been evaluated by Tehrany, Pradhan & Jebur (2013) in Kelantan, Malaysia. The prediction accuracy of their results proved the proficiency of this method in flood studies. The drawback of using the DT method is the considerable amount of time required to produce the final tree. SVM is a powerful machine learning technique in probability analysis. Using this technique, pixels can be categorized even when the data are not linearly separable. Its processing speed varies based on the selected SVM kernel.

Similar to other natural disasters, floods cause costly and irrecoverable damages to the affected areas. Although it is almost impossible to prevent flooding, high-risk areas can be recognized, and the damages can be considerably reduced by proper management. Appropriate planning and management can be carried out by performing flood susceptibility, hazard, and risk analyses. The areas that are susceptible to floods must be detected; the accuracy of the outcomes is directly associated with the efficiency of the technique used and the accuracy of the dataset used.

Based on the aforementioned literature, there is a lack of optimized techniques for obtaining flood susceptibility maps. In addition, there are several methods such as EBF that have not yet been used for obtaining these maps. The idea of creating a more reliable method by combining two or more techniques may resolve the issues involved in the individual methods (Rokach, 2010). It has been shown by several researchers that an ensemble technique is more efficient in terms of prediction accuracy than individual methods (Lee & Oh, 2012). A recent study that was implemented by Choubin et al. (2019) has proved the proficiency of ensemble modeling in flood analysis. They used three methods for performing the analysis; these include multivariate discriminant analysis, classification and regression trees, and SVM. Tehrany et al. (2014) resolved the issues faced by FR and LR in flood susceptibility mapping by integrating both the methods. A similar method has been tested by Umar et al. (2014) to map landslide-susceptible regions in West Sumatera Province, Indonesia. Although several methods and their applications to flood susceptibility mapping have been examined, an ensemble analysis that includes the integration of EBF and SVM has not been tested for this purpose. The reasons that these methods were chosen are as follows:

1. Four SVM Kernels (linear (LN), polynomial (PL), radial basis function (RBF), and sigmoid (SIG)) provide more detail in the assessment and reliability of the derived ensemble method. If all the kernels in the ensemble method provide a higher accuracy than the individual methods, it proves the proficiency of the ensemble method. Every method does not provide the opportunity to not only evaluate the outcomes using an accuracy assessment technique but also using various internal factors.
2. The study conducted by Fotovatikhah et al. (2018) investigated more than hundred articles on floods. According to their results, SVM methods exhibited lower error rates in comparison with those exhibited by other methods.
3. The EBF method has been rarely used in flood susceptibility mapping, but its application has been repeatedly examined in other natural hazard domains.
4. EBF is a robust method based on the Dempster-Shafer theory in which relative flexibility is one of the benefits. It is capable of generating reliable outcomes by integrating different factors to decrease the uncertainty (Thiam, 2005). This technique assesses the likelihood that a certain theory is correct, and it estimates how closely the proof confirms the correctness of that hypothesis. The degree of belief (*Bel*), degree of uncertainty (*Unc*), degree of disbelief (*Dis*), and degree of plausibility (*Pls*) are the main parameters of EBF each of which extracts specific information using different analysis of a dataset.
5. A combination of SVM and EBF is an integration of a powerful machine learning method and a strong statistical method, respectively.

An individual SVM and the application of its four kernels in flood susceptibility mapping has been tested by Tehrany et al. (2015). However, EBF is almost new in the flood susceptibility domain. EBF is mostly used in mineral potential mapping (Carranza, 2009; Ford, Miller & Mol, 2015), landslide mapping (Althuwaynee, Pradhan & Lee, 2012; Lee, Hwang & Park, 2013), land subsidence mapping (Pradhan et al., 2014), forest fire susceptibility mapping (Pourghasemi, 2016), and groundwater mapping (Nampak, Pradhan & Manap, 2014; Pourghasemi & Beheshtirad, 2015). The aim of this study is to enhance the prediction accuracy of individual SVM and EBF methods in flood susceptibility mapping by combining them. The SVM and EBF methods are applied individually to compare the performance of the new ensemble method with that of the individual ones. In addition, all the SVM kernel types are used in the ensemble modeling because each has a specific analysis process that produces a different outcome. A comparison between the SVM kernels can assist in identifying the most proficient method for natural hazard studies. Finally, the precision and reliability of the results are assessed using the area under the curve (AUC) method.

## Study area and data

**Figure 1:**
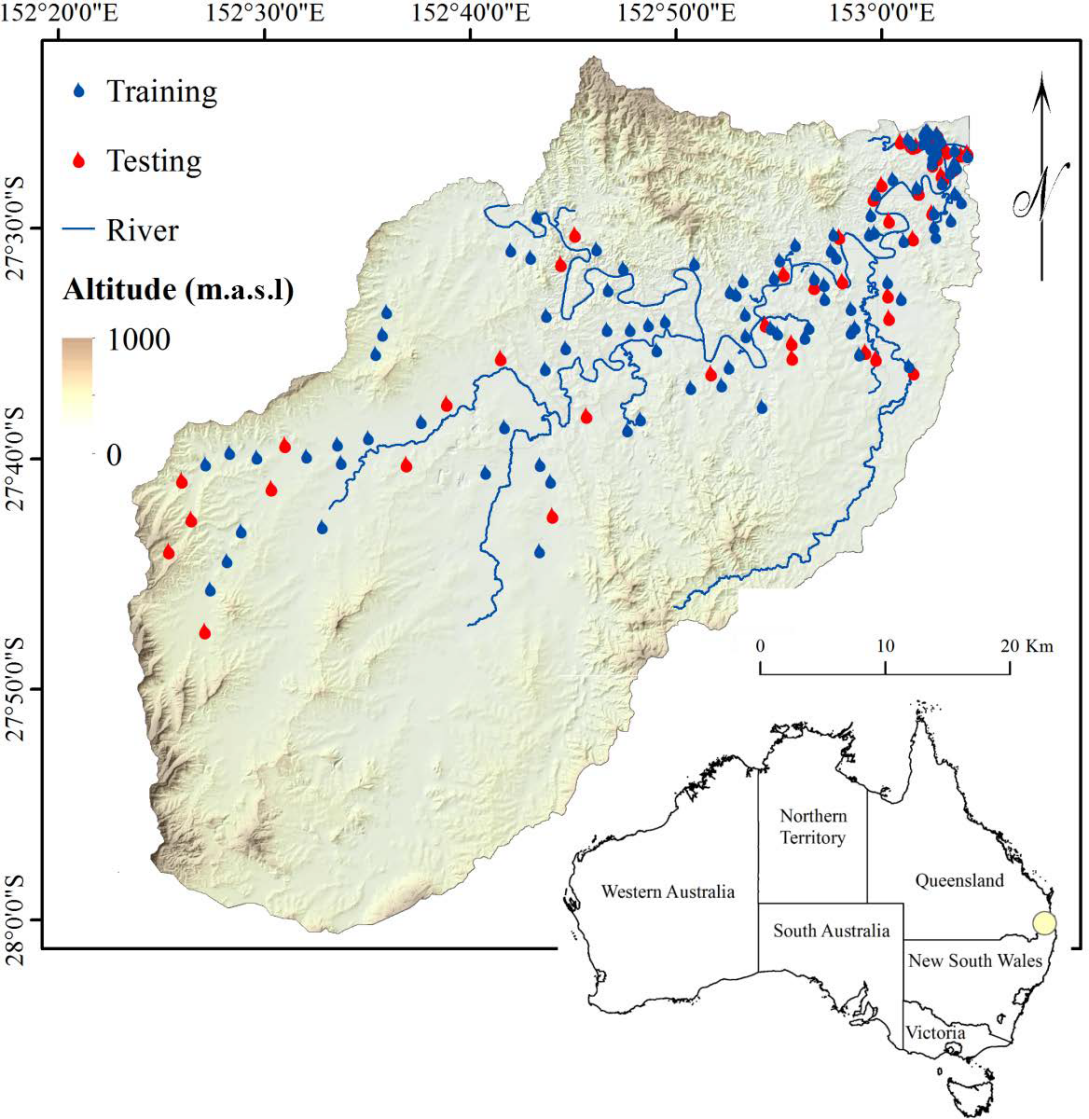
Study area and inundated points used as inventory data in this research.

The Brisbane River Catchment in Queensland, Australia, was chosen as the study area. Three sub-basins of the Bremer River, Brisbane River, and a part of Lockyer Creek are covered by this catchment. The study area has an approximate area of 2,806 km^2^ located between latitudes 27°22′12″S and 28°01′48″S and longitudes 152°22′12″E and 153°05′6″E (Fig. 1). In Queensland, the average yearly precipitation ranges from very low values in the Southwest to very high values exceeding 2,000 mm around the coastal regions. Even in regions with low precipitation, considerably heavy rainfall takes place in some years, thereby causing floods. Scientists believe that long-term climate change may affect rainfall in this region (Partridge, 2001). Brisbane has a humid subtropical climate with very hot, humid summers and dry, reasonably warm winters. The average temperature is 20.3 °C, and it receives nearly 1,168 mm of rainfall per year. A destructive flood occurred in Brisbane in 2001 and is used in this study as inventory data. The flood forced the evacuation of many people from towns and cities. Vast areas around the Brisbane River were inundated, and there was significant damage and loss of life.

To perform flood susceptibility mapping, two sets of data are required. The first dataset represents the historical data of floods that indicates the inundated regions (a flood inventory map). The second dataset is related to flood contributing parameters that are known as flood conditioning factors (Lee, Hwang & Park, 2013). Flood inventory data need to be assessed against flood conditioning factors to recognize their significance and impact on the occurrence of the floods because it is typically assumed that floods will occur under the same conditions as before (Fotovatikhah et al., 2018). Then, the inventory data must be divided into the training and testing datasets to be used for the training and validation processes, respectively (Tsangaratos & Ilia, 2016). In flood modeling, there is no specific or pre-defined method that exists for classifying inventory data. It is typically decided based on the accessibility and quality of data. Space robustness and time robustness are two measures used for assessment (Althuwaynee et al., 2014a). In time robustness, flood inventory data are split into two periods: past incidence that represent the training data, and future incidence that represent the validation data. Multi-temporal data are required for this analysis, wherein each flood is associated with the precipitation data that caused it. In space robustness, flood inventory data are randomly split into two classes: training and testing. When comprehensive flood inventory data are available, integration of these methods is possible (Huabin et al., 2005). In this study, the space robustness technique was used to generate the training and testing datasets.

These training and testing datasets were also used later in the validation stage (Xu, Xu & Yu, 2012). The validation process was implemented by comparing the existing flood locations with the acquired flood susceptibility map. The AUC method, which is described in the methodology section (‘Validation’), was used to assist the validation. The success and prediction rates of the AUC method were measured using the training and testing datasets, respectively. The success rate represented how well the model fit to the training dataset (Tehrany et al., 2015). The prediction capability of the model cannot be assessed by the success rate because it is measured using the flood locations that have already been used for constructing the model. The prediction rate can be used to evaluate the prediction capability of the model. The prediction rates were measured by comparing the flood susceptibility maps with the flood testing dataset (Bui et al., 2012).

According to the literature, the percentages commonly used to divide the inventory dataset are 30% and 70% for the testing and training datasets, respectively (Abdulwahid & Pradhan, 2017; Chen et al., 2019; Pham et al., 2017a). In the study conducted by Kalantar et al. (2018), the impacts of training data selection on the susceptibility mapping have been evaluated.

From 159 flood locations, 106 locations were used for the purpose of training, and the remaining 53 locations were used for validation (Fig. 1). With regard to the data configuration, specific data preparation was required. Two separate data layers were created for training and testing. The training flood locations (106 points) were selected randomly to produce the dependent data consisting of values 0 and 1 that represent the existence and absence of flooding over a region, respectively. The same number of points (106) were selected as non-flooded areas, and value 0 was assigned to them. Considering the non-flooded locations in the study area can enhance the accuracy of the results (Tehrany et al., 2015). The rest of the flood events (53 points) were used for the purpose of testing. The same configuration was also used to create the testing data layer.

**Figure 2:**
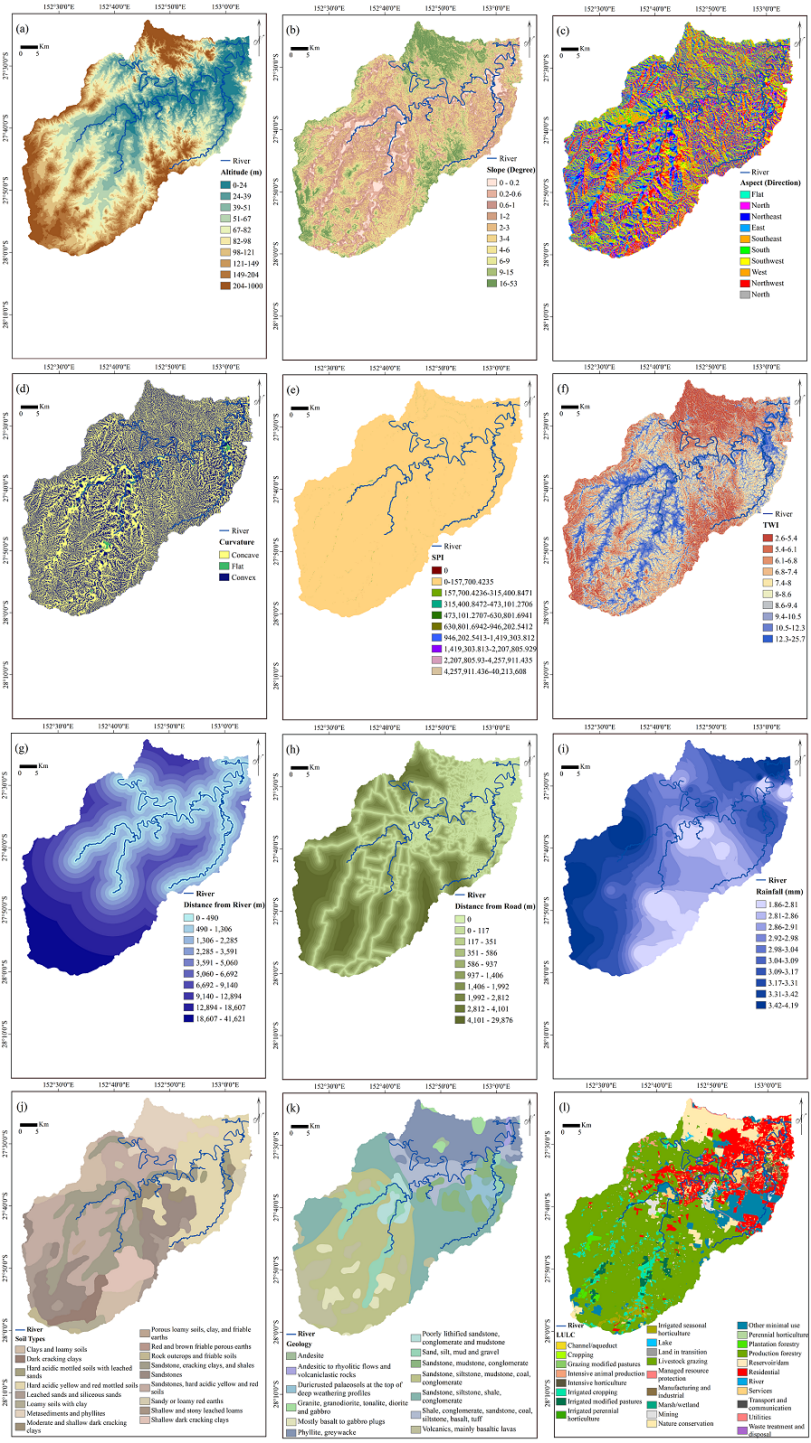
Flood conditioning factors. (A) Altitude, (B) slope, (C) aspect, (D) curvature, (E) stream power index (SPI), (F) topographic wetness index (TWI),(G) distance from rivers, (H) distance from roads, (I) rainfall, (J) soil types, (K) geology, (L) land use land cover (LULC).

In terms of flood conditioning factors, the selection of the most influential parameters is essential. Precipitation is the most significant parameter in the occurrence of floods. However, many other parameters are involved (Lawal et al., 2012). Flooding is initiated by rainfall but influenced by many other factors. During rainfall in a drainage basin, the extent of rain that enters the rivers depends on the condition of the basin, mainly its extent, topography, and LULC types (Hölting & Coldewey, 2019). Some rainfall is controlled by vegetation and soil, and the remaining rainfall reaches the rivers. Twelve flood conditioning factors (slope, aspect, elevation, curvature, topographic wetness index (TWI), geology, stream power index (SPI), soil, LULC, rainfall, distance from roads, and distance from rivers) were collected from different sources and converted into a raster format with a 5 × 5 m pixel size (Table 1). All the scale factors were classified using the quantile method, and they are presented in Fig. 2. The factors were classified because EBF is a BSA method that assesses the influence of each class of a conditioning factor on a specific event, which, in the current case, is floods.

**Table 1:** Spatial dataset and data sources.

**Conditioning factor**	**Source**	**Impact**
Altitude	Light Detection and Ranging (LiDAR) data from Australian Government/Geoscience Australia	High-elevation regions help water flow and connect to lower areas around the rivers, causing flooding.
Slope	Derived from DEM	Impact on the extent and velocity of runoff.
Aspect	Derived from DEM	Effect on the amount of precipitation and sunshine.
Curvature	Derived from DEM	Influence on surface infiltration.
SPI	Derived from DEM	Erosive power of the terrain.
TWI	Derived from DEM	Amount of the flow accumulation at any place in a catchment.
Soil	CSIRO website	Soil type and soil structure control the soil saturation and amount of water infiltration in soil.
Geology	Queensland Government website	Impact on rainfall penetration and water flow.
LULC	• Classifying SPOT5 imagery	Each LULC type plays specific role in flooding.
	• High spatial resolution orthophotography	
	• Scanned aerial photos	
	• Local expert knowledge	
Rainfall	Bureau of Meteorology website	Floods occur after heavy precipitation.
Distance from river	Queensland government website (Wetlandinfo)	Areas closer to the rivers have higher chance of getting flooded.
Distance from road	Department of Transport and Main *Roads*	Impervious surfaces produce more flooding.

Floods typically occur in regions with low elevation (Botzen, Aerts & Van den Bergh, 2013). Water moves from the hillsides of mountains and reaches the lower ground; this leads to flooding. Researchers consider the altitude an amplifying parameter in the occurrence of floods because it has an impact on the amount and velocity of runoff (Kia et al., 2012). Altitude and its derivatives have vital roles in identifying areas that are susceptible to flooding. More reliable flood analysis can be expected when more accurate topographical data are used (Abdullah, Vojinovic & Rahman, 2013). A DEM with a spatial resolution of 5 m that was produced from Light Detection and Ranging (LiDAR) data was used to derive other related parameters. Slope layer, another topographical parameter, was produced from DEM with 10 classes with a maximum angle of 53°. The slope impact on flooding is related to runoff speed: steep slopes have less time for infiltration, which causes an increase in water flow. An aspect map that has nine classes indicating the direction of the terrain (flat, northeast, east, southeast, south, southwest, west, and northwest) was also derived from DEM. The curvature (slope shape) has three classes: concave (positive values (+)), convex (negative values (−)) and flat (value 0). Water-associated parameters of TWI and SPI were also used in the analysis, and they were measured using the following equations (Tehrany, Pradhan & Jebur, 2014):

(1) \begin{eqnarray*}& & \mathrm{TWI}=\ln \nolimits \left( {A}_{s}/\tan \nolimits \beta \right) \end{eqnarray*}

(2) \begin{eqnarray*}& & \mathrm{SPI}={A}_{s}\tan \nolimits \beta \end{eqnarray*}

where *A*_*s*_ is the area of catchment (m^2^) and *β* (radians) is the slope gradient.

Although both TWI and SPI factors have been derived from the catchment area and slope, each represents different terrain characteristics. SPI measures the erosive power of flowing water (Althuwaynee et al., 2014b). It is expected that flooding occurs in the areas with the lowest SPI values, the reason being that most areas with high SPI values are sharp and steep lands. Therefore, gravity increases the speed of water flow; consequently, destructive power increases. On the other hand, the spatial distribution and zone of saturation of sources for runoff generation can be identified by measuring the TWI. The TWI is used to measure topographic control on hydrological procedures (Chen & Yu, 2011). It shows the water penetration capability in a region and thus, the areas with potential for floods. Logically, flat terrain absorbs more water than steep terrain owing to more gravity acting on the water flowing down the hilly slopes. Hence, the TWI in areas around rivers and flat lands is greater than that in areas with slopes. Higher TWI values are usually found in flooded areas.

The distance from a river and the distance from a road were determined using the Euclidean Distance tool, and ten classes were created for each parameter. Urbanization increases the areas with impervious surfaces that cause increased hydraulic proficiency in urban basins. Hence, the terrain has less rainfall infiltration capacity that increases the extent of runoff (Shuster et al., 2005). Owing to the significant role of LULC, this factor was also used in the analysis. Different soil conditions can affect the extent of runoff in the catchment area. Some soil types allow greater infiltration of precipitation compared to others, which leads to a smaller volume of runoff. Different types of geology can also affect the amount and speed of water flow.

## Methodology

**Figure 3:**
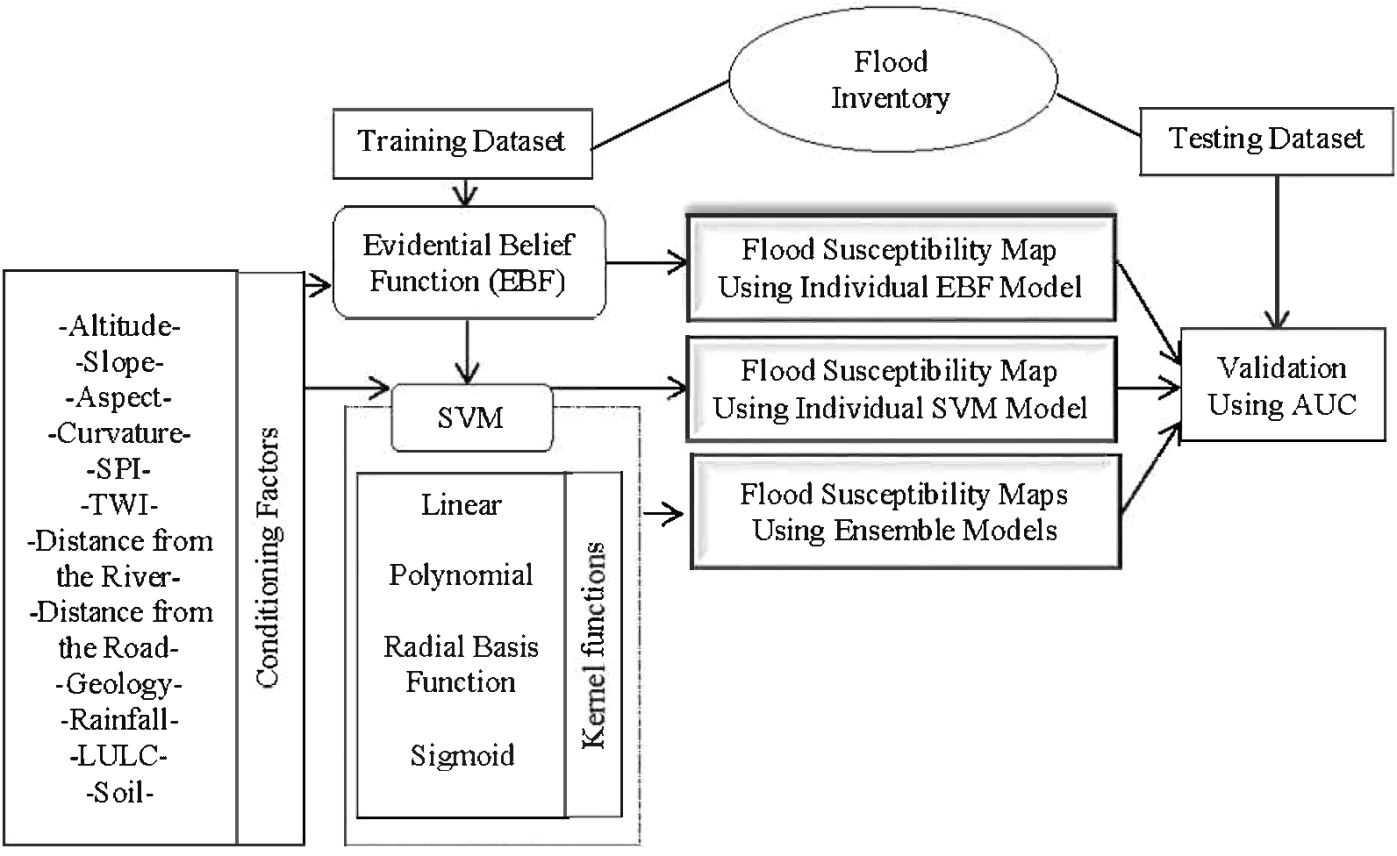
Methodology flowchart.

The process commenced by performing EBF using the flood training points. The correlation between each class of a conditioning factor and flood occurrence was assessed. All the factors were reclassified using the derived weights and used in SVM analysis as inputs. SVM analysis was performed using all the four kernels (LN, PL, SIG, and RBF) because each kernel has a different method of analysis. To clearly judge the performance of the ensemble methods, EBF and SVM were also applied individually. All six derived susceptibility maps were validated using the AUC technique and the flood testing dataset. The procedure is shown in Fig. 3.

### Evidential belief function (EBF)

The Dempster–Shafer technique is a statistical procedure that is used to recognize spatial integration between dependent and independent factors (Smets, 1994). The Dempster-Shafer theory (DST) of evidence, developed by Dempster (2008), is a generalization of the Bayesian theory of subjective probability. Its major advantages are its relative flexibility in accepting uncertainty and the ability to combine beliefs from multiple sources of evidence (Tehrany et al., 2017).

Suppose that a set of flood conditioning factors *C* = (*C*_*i*_, *i* = 1, 2, 3, …, *n*) that includes mutually exclusive and exhaustive factors of *C*_*i*_ is used in this research. *C* is known as the frame of discernment. A basic probability assignment is a function *m*: $P(C)\rightarrow \left[ 0,1 \right] .P(C)$ is the set of all subsets of *C* including the empty set and *C* itself. This function is also called a mass function and satisfies $m \left( \Phi \right) =0$ and ${\sum }_{AC}m \left( A \right) =1$, where Φ is an empty set and *A* is any subset of *C*. $m \left( A \right) $ measures the degree to which the evidence supports *A*, and it is denoted by *Bel* (*A*), a belief function. The degree of belief (*Bel*), degree of uncertainty (*Unc*), degree of disbelief (*Dis*), and degree of plausibility (*Pls*) are the main parameters of EBF (Althuwaynee, Pradhan & Lee, 2012). The dissimilarity among *Bel* and *Pls* is represented by *Unc*, which represents ignorance. *Dis* is the degree of belief of the hypothesis being incorrect for certain evidence. The relationships between these parameters have been previously described, and they include 1 − *Unc* − *Bel*, *Dis* = 1 − *Pls* and *Bel* + *Unc* + *Dis* = 1, where *C*_*ij*_ has no flood event, *Bel* will be zero, and *Dis* will be reset to zero (Awasthi & Chauhan, 2011).

Both BSA and MSA can be performed using EBF (Carranza, Woldai & Chikambwe, 2005). The number of pixels that represent flood or non-flood for each class of a flood conditioning factor are measured by overlapping the flood inventory layer of all flood conditioning factors. Assuming that *N*(*L*) and *N*(*C*) are the pixels that are inundated and that *C*_*ij*_ is the *j*th class of the flood contributing factor *C*_*i*_(*i* = 1, 2, 3, …, *n*), *N*(*C*_*ij*_) is the number of pixels in class *C*_*ij*_, and *N* = (*L*∩*C*_*ij*_) is the number of inundated pixels in *C*_*ij*_. Hence, EBF can be calculated as follows (Carranza & Hale, 2003):

(3) \begin{eqnarray*}& & Bel \left( {C}_{ij} \right) = \frac{{W}_{{C}_{ij}(\text{Flood})}}{\sum _{j=1}^{n}{W}_{{C}_{ij}(\text{Flood})}} \end{eqnarray*}

(4) \begin{eqnarray*}& & {W}_{{C}_{ij}(\text{Flood})}= \frac{ \frac{N(L\cap {C}_{ij})}{N(L)} }{ \frac{[N({C}_{{}_{ij}})-N(L\cap {C}_{ij})]}{[N(C)-N(L)]} } \end{eqnarray*}

(5) \begin{eqnarray*}& & Dis \left( {C}_{ij} \right) = \frac{{W}_{{C}_{ij}(\text{Non-flooded})}}{\sum _{j=1}^{n}{W}_{{C}_{ij}(\text{Non-flooded})}} \end{eqnarray*}

where

(6) \begin{eqnarray*}& & {W}_{{C}_{ij}\text{(Non-flooded)}}= \frac{ \frac{[N \left( {C}_{ij} \right) -N(L\cap {C}_{ij})]}{N(L)} }{ \frac{[N(C)-N \left( L \right) -N \left( {C}_{ij} \right) +N(L\cap {C}_{ij})]}{[N(C)-N(L)]} } .\end{eqnarray*}

The numerator in Eq. (4) is the proportion of flooded pixels in factor class *C*_*ij*_; the numerator in Eq. (6) is the proportion of flooded pixels that do not occur in factor class *C*_*ij*_; the denominator in Eq. (4) is the proportion of non-flooded pixels in factor class *C*_*ij*_; the denominator in Eq. (6) is the proportion of non-flooded pixels in other attributes outside the factor class *C*_*ij*_. Here, the weight of *C*_*ij*_ is represented by *W*_*C*_*ij*_(Flood)_, which supports the belief that floods are more likely to occur, and *W*_*C*_*ij*_(Non-flood)_ represents the weight of *C*_*ij*_ that supports the belief that floods are less likely to occur.

Excel and ArcGIS software were used to measure the EBF. Subsequently, Dempster’s rule of combination was applied using a raster calculator in ArcGIS to obtain the four integrated EBFs. The formulae for combining two flood conditioning factors *C*_1_ and *C*_2_ are as follows:

(7) \begin{eqnarray*}& & {Bel}_{{C}_{1}{C}_{2}}= \frac{{Bel}_{{C}_{1}}{Bel}_{{C}_{2}}+{Bel}_{{C}_{1}}{Unc}_{{C}_{2}}+{Bel}_{{C}_{2}}{Unc}_{{C}_{1}}}{1-{Bel}_{{C}_{1}}{Dis}_{{C}_{2}}-{Dis}_{{C}_{1}}{Bel}_{{C}_{2}}} \end{eqnarray*}

(8) \begin{eqnarray*}& & {Dis}_{{C}_{1}{C}_{2}}= \frac{{Dis}_{{C}_{1}}{Dis}_{{C}_{2}}+{Dis}_{{C}_{1}}{Unc}_{{C}_{2}}+{Dis}_{{C}_{2}}{Unc}_{{C}_{1}}}{1-{Bel}_{{C}_{1}}{Dis}_{{C}_{2}}-{Dis}_{{C}_{1}}{Bel}_{{C}_{2}}} \end{eqnarray*}

(9) \begin{eqnarray*}& & {Dis}_{{C}_{1}{C}_{2}}= \frac{{Dis}_{{C}_{1}}{Dis}_{{C}_{2}}+{Dis}_{{C}_{1}}{Unc}_{{C}_{2}}+{Dis}_{{C}_{2}}{Unc}_{{C}_{1}}}{1-{Bel}_{{C}_{1}}{Dis}_{{C}_{2}}-{Dis}_{{C}_{1}}{Bel}_{{C}_{2}}} \end{eqnarray*}

Integrated EBF of the flood conditioning factors are implemented sequentially using Eqs. (7)–(9).

### Support vector machine (SVM)

Among the data-driven techniques, machine learning methods produce promising viewpoints in natural hazard mapping, and they are suitable for nonlinear multi-dimensional modeling problems (Yilmaz, 2010). SVM is based on the statistical learning concept. It contains a stage wherein the model is trained using a training dataset of related input and target output values. After the model is trained, it is used to assess the testing data. There are two main procedures underlying SVM for solving problems (Yao, Tham & Dai, 2008). First, a linear separating hyper-plane is created that splits the data based on their patterns. Second, mathematical functions (kernels) are used to transform the nonlinear data into a linearly distinguishable format (Micheletti et al., 2011).

Separating hyper-plane formations from a training dataset is the basis for this method. The separated hyper-plane is generated in the original space of *n* coordinates (*x*_*i*_: parameters of vector *x*) between the points of two distinct classes (Shao & Deng, 2012). Values of +1 and −1 are assigned to the pixels that are above and below the hyper-plane, respectively. The training pixels that are closest to the hyper-plane are called support vectors. Modeling of the rest of the data can be undertaken after deriving the decision surface (Pradhan, 2013). The maximum margin of separation between the classes is discovered by SVM; therefore, it builds a classification hyper-plane in the center of the maximum margin.

Consider a training dataset of instance-label pairs (*x*_*i*_, *y*_*i*_) with *x*_*i*_ ∈ *R*^*n*^, *y*_*i*_ ∈ {1, −1} and *i* = 1, …, *m*. In this case study, *x* represents slope, aspect, elevation, curvature, TWI, geology, SPI, soil, LULC, rainfall, distance from roads, and distance from rivers. The classes of 1 and −1 show the flooded and non-flooded pixels, respectively. Finding the best hyper-plane is the goal of the SVM, which separates pixels into different classes, namely, flooded and non-flooded. A separating hyper-plane can be defined as:

(10) \begin{eqnarray*}& & {y}_{i} \left( w.{x}_{i}+b \right) \geq 1-{\xi }_{i},\end{eqnarray*}

where the orientation of the hyper-plane in the feature space is shown by *w*, the offset of the hyper-plane from the origin is represented by *b*, and the positive slack variable is *ξ*_*i*_ (Cortes & Vapnik, 1995). The following optimization problem using Lagrangian multipliers was solved through the determination of an optimal hyper-plane (Samui, 2008).

(11) \begin{eqnarray*}& & \mathrm{Minimize}\quad \sum _{i=1}^{n}{\alpha }_{i}- \frac{1}{2} \sum _{i=1}^{n}\sum _{j=1}^{n}{\alpha }_{i}{\alpha }_{j}{y}_{i}{y}_{j} \left( {x}_{i}{x}_{j} \right) ,\end{eqnarray*}

(12) \begin{eqnarray*}& & \mathrm{subject~ to}\quad \sum _{i=1}^{n}{\alpha }_{i}{y}_{j}=0,\quad 0\leq {\alpha }_{i}\leq C,\end{eqnarray*}

where *α*_*i*_ are Lagrange multipliers, *C* is the penalty, and the slack variables *ξ*_*i*_ allow the penalized constraint violation. Then, the decision function that is used to classify the new data can be written as:

(13) \begin{eqnarray*}& & g \left( x \right) =\mathrm{sign} \left( \sum _{i=1}^{n}{y}_{i}{\alpha }_{i}{x}_{i}+b \right) .\end{eqnarray*}

In the case where the hyper-plane cannot be separated by the linear kernel function, the original input data may be shifted into a high-dimension feature space through some nonlinear kernel functions. Then, the classification decision function is written as (Pradhan, 2013):

(14) \begin{eqnarray*}& & g \left( x \right) =\mathrm{sign} \left( \sum _{i=1}^{n}{y}_{i}{\alpha }_{j}K \left( {x}_{i},{x}_{j} \right) +b \right) \end{eqnarray*}

where *K*(*x*_*i*_, *x*_*j*_) is the kernel function.

All of the conditioning factors were reclassified using the obtained EBF weights and entered into SPSS Modeler to implement the SVM modeling. Selection of the kernel function is very important in SVM modeling (Pradhan, 2013). SPSS Modeler offers four types of SVM kernels: LN, PL, RBF, and SIG. RBF is the most popular kernel because it works well in most cases (Yao, Tham & Dai, 2008). RBF has high interpolation capability and less extrapolation capability (Kavzoglu & Colkesen, 2009). PL has an inverse situation, which has better extrapolation capabilities compared to RBF. SIG and RBF perform in a similar manner for certain parameters. However, RBF offers more accuracy (Song et al., 2011). The LN kernel is less popular because it is based on a linear assumption. Using different kernels results in different outcomes. Therefore, in this study, all the kernels were used in ensemble modeling to find the optimal results and compare the outputs. The mathematical representation of each kernel is listed below (Pourghasemi et al., 2013a):

where *γ* (gamma) is a common parameter for all kernels except LN; *d* shows the polynomial degree term in the polynomial kernel function; *r* represents the bias term in the polynomial and sigmoid kernel functions. The parameters *γ*, *d* and *r* are defined by the user. The accuracy of these parameters directly influences the reliability and correctness of SVM outcomes (Ballabio & Sterlacchini, 2012). The two other SVM parameters of the cost of constraint violation (*C*) and epsilon (ε) were considered constant throughout the analysis.

Because of the importance of kernel parameter selection, listed in Table 2, a cross-validation method was used instead of the trial and error method (Zhuang & Dai, 2006). This process commenced with the division of the flood inventory into *n* folds: one fold was kept for accuracy assessment purposes, and the rest were saved to run the model (Yao, Tham & Dai, 2008). In this study, the dataset was split into five random folds for which every group had an equal number of flood points (Table 3). The average of the parameters was used for the final training.

**Table 2:** Different SVM kernel types, their equations and required parameters.

**Kernel**	**Equation**	**Kernel parameters**
RBF	$K \left( {x}_{i},{x}_{j} \right) =\mathrm{exp} \left( -\gamma \parallel {x}_{i}-{x}_{j}{\parallel }^{2} \right) $	*γ*
LN	$K \left( {x}_{i},{x}_{j} \right) ={x}_{i}^{T}{x}_{j}$	–
PL	$K \left( {x}_{i},{x}_{j} \right) =(-\gamma {x}_{i}^{T}x+r)^{d}$	*γ*, *d*
SIG	$K \left( {x}_{i},{x}_{j} \right) =\mathrm{Tanh}(-\gamma {x}_{i}^{T}x+r)^{d}$	*γ*

**Table 3:** Cross-Validation results.

EBF & RBF-SVM Model	Training fold	Testing fold	*γ*		*C*
1	2, 3, 4, 5	1	0.1		20
2	1, 3, 4, 5	2	0.2		10
3	1, 2, 4, 5	3	0.1		10
4	1, 2, 3, 5	4	0.3		12
5	1, 2, 3, 4	5	0.2		15
			0.18		13.5
EBF & SIG-SVM Model	Training fold	Testing fold	*γ*		*C*
1	2, 3, 4, 5	1	2		20
2	1, 3, 4, 5	2	1.5		10
3	1, 2, 4, 5	3	1		10
4	1, 2, 3, 5	4	2		10
5	1, 2, 3, 4	5	3		10
			1.9		12
EBF & LN-SVM Model	Training fold	Testing fold	*C*		
1	2, 3, 4, 5	1	10		
2	1, 3, 4, 5	2	11		
3	1, 2, 4, 5	3	14		
4	1, 2, 3, 5	4	15		
5	1, 2, 3, 4	5	15		
			13		
EBF & PL-SVM Model	Training fold	Testing fold	*γ*	*d*	*C*
1	2, 3, 4, 5	1	1	3	10
2	1, 3, 4, 5	2	1	3	20
3	1, 2, 4, 5	3	5	5	20
4	1, 2, 3, 5	4	5	1	10
5	1, 2, 3, 4	5	10	7	10
			4.4	3.8	14

### Ensemble modeling

To perform the ensemble modeling, all the flood conditioning factors were reclassified based on the acquired EBF weight of *C*_*ij*_. This stage represents the BSA. The next stage denotes the MSA by reclassifying the conditioning factors using the derived weights from EBF and using them in the SVM analysis. The ensemble method was applied using all four SVM kernels and the parameters obtained from the cross-validation. Consequently, four flood probability indices were derived. In addition, another flood probability map was generated using an individual SVM and an RBF kernel. A stand-alone SVM analysis was performed using the original flood conditioning factors that were not classified by the EBF results. The conditioning factors used in the individual SVM modeling were unclassified and were all in a continuous data format. The reason for this was to examine whether the data format or the use of classified factors can reduce the data variability (Sajedi Hosseini et al., 2018). In addition, individual EBF results were modeled, and a flood probability index was calculated.

### Spatial sensitivity analysis

Uncertainty is an unavoidable factor in every analysis (Rahmati, Pourghasemi & Melesse, 2016). Considering these uncertainties helps in obtaining better interpretations of the model outcomes. Although it is not possible to achieve 100% accuracy, there are several approaches that can be implemented to reduce the uncertainty (Refsgaard et al., 2007). These include uncertainty engine (Brown & Heuvelink, 2007), inverse modeling (predictive uncertainty) (Friedel, 2005), and Monte Carlo analysis (Yang, 2011). Sensitivity analysis (SA) evaluates the impact of conditioning factor variations on model outputs, thereby allowing the quantitative assessment of the relative importance of uncertainty sources (Chen, Yu & Khan, 2010). In this study, the Jackknife test was used to assess the uncertainty among the conditioning factor datasets. This SA technique examines the impact of repeatedly removing every conditioning factor from the dataset on the final outcomes. This means that by using this process, the conditioning factor contribution in the analysis can be recognized. In addition, the percentage of relative decrease (PRD) of the AUC values was measured to investigate the dependency of the model output on the influence of conditioning factors using the following equation:

(15) \begin{eqnarray*}& & \mathrm{PRD}= \frac{{\mathrm{AUC}}_{\mathrm{all}}-{\mathrm{AUC}}_{i}}{{\mathrm{AUC}}_{i}} \times 100\end{eqnarray*}

where AUC_all_ indicates the AUC value derived using the full conditioning factor dataset, and AUC_*i*_ shows the prediction power of the method when the *i* th conditioning factor has been excluded from the dataset.

### Validation

To evaluate the efficiency and reliability of the analytical outcomes, the popular AUC method was used. AUC is a popular assessment technique in natural hazard analysis because it provides an understandable and comprehensive way for performing validation (Beguería, 2006; Hand & Till, 2001). It commences with the arrangement of the probability index in descending order. Classification of the probability index into hundred categories on the *y*-axis with cumulative 1% breaks is the second step. Then, the flood occurrence in each class is examined, and prediction and success rates are derived. The prediction rate is the accuracy that is achieved using flood testing points. It shows how successful the applied technique was in predicting the flood-prone areas that were already inundated. Conversely, the success rate is produced using the flood training points, and this shows the model performance (Tehrany et al., 2015). The range of the AUC is between zero and one. The maximum accuracy is represented by the value 1, and 0 indicates the failure of the analysis. In this study, 106 flood locations were used for training and 53 locations were used for testing purposes.

## Results and Discussion

### Analyzing the weights derived from each method

EBF was applied, and the weight for each class of the flood conditioning factors was determined. The areas with high values of *Bel* and low values of *Dis* are the most susceptible to floods. Table 4 lists the EBF calculated for the twelve flood parameters. A range of 0.22 to 23.75 m in altitude received the highest *Bel* (77) and lowest *Dis* (2) values, thereby indicating the highest susceptibility to floods. All the altitude classes except the second one had considerably low *Bel* values, thereby indicating low susceptibility to floods. EBF results acquired for altitudes confirmed that most flooding occurred at low altitudes because the water flowed to and met in the lower areas, thereby indicating that flooding of areas at higher altitudes is almost impossible. The correlation between landslide occurrence and slope shows that steep slopes accelerated water flows. A range of 0–0.21° in the slope map attained the highest *Bel* value of 29 and a low *Dis* value of 8, followed by the slope range 0.62–1.25°. The aspect map received the highest *Bel* value of 52 and the lowest value of 8 for the class that was flat; this shows that floods occur in flat areas because water cannot infiltrate the saturated soil. Moisture preservation and vegetation density are affected by this aspect, which also influences flood occurrence. The morphology of the topography is indicated by curvature, which has three categories: concave, convex, and flat. A pixel with a negative curvature value denotes upward concave ground; a pixel with a positive curvature value denotes upward convex ground. A pixel with value zero represents flat ground. The *Bel* values for the convex and concave categories in the curvature map were low; this condition implies lower flood potential compared with the flat curvature class. The flat class received *Bel* and *Dis* values of 54 and 17, respectively.

**Table 4:** Results of EBF in the case of each factor.

Layer	Classes	Pixels in class	Pixels in domain	*Bel*	*Dis*
Elevation (m)	0–23.75	1,214,340	81	77	2
	23.75–39.43	1,221,749	12	11	9
	39.43–51.19	1,427,269	4	3	10
	51.19–66.87	1,474,786	1	0	11
	66.87–82.56	1,480,835	4	3	10
	82.56–98.24	1,010,034	3	3	10
	98.24–121.76	1,384,453	1	0	11
	121.76–149.21	1,049,967	0	0	10
	149.21–204.10	1,151,800	0	0	11
	204.10–1000.01	1,056,388	0	0	10
Slope	0–0.21	726,676	19	29	8
	0.21–0.62	1,343,717	18	15	9
	0.62–1.25	1,571,205	30	21	8
	1.25–2.09	1,449,461	18	14	9
	2.09–3.13	1,345,964	10	8	10
	3.13–4.39	1,240,403	3	2	10
	4.39–6.27	1,289,769	5	4	10
	6.27–9.41	1,214,967	1	0	10
	9.41–15.05	1,147,790	1	0	10
	15.05–53.32	1,141,669	1	1	10
Aspect	Flat	498,560	29	52	8
	North	1,593,563	12	6	11
	Northeast	1,708,517	10	5	11
	East	1,813,190	10	4	11
	Southeast	1,527,816	9	5	11
	South	1,216,894	9	6	11
	Southwest	1,147,405	10	7	11
	West	1,391,379	7	4	11
	Northwest	1,574,297	10	5	11
Curvature	Convex	141,143	1	45	40
	Flat	12,201,477	105	54	17
	Concave	129,001	0	0	41
SPI	0	2,479	0	0	11
	0–157700.42	2,479	0	0	11
	157700.42–315400.84	12,450,355	106	100	0
	315400.84–473101.27	10,969	0	0	11
	473101.27–630801.69	3,631	0	0	11
	630801.69–946202.54	1,609	0	0	11
	946202.54–1419303.81	1,030	0	0	11
	1419303.81–2207805.92	595	0	0	11
	2207805.92–4257911.43	367	0	0	11
	4257911.43–40213608	311	0	0	11
TWI	2.595171–5.410969	1,110,528	1	1	10
	5.410969–6.137627	1,289,883	0	0	11
	6.137627–6.773453	1,280,133	1	0	11
	6.773453–7.409278	1,399,032	6	4	10
	7.409278–8.045104	1,374,035	3	2	10
	8.045104–8.680929	1,199,716	9	8	10
	8.680929–9.498419	1,238,009	14	12	9
	9.498419–10.588406	1,214,072	22	20	8
	10.588406–12.314218	1,215,006	18	16	9
	12.314218–25.757385	1,151,207	32	31	7
Soil	Metasediments and phyllites	896,047	1	1	6
	Hard acidic yellow and red mottled soils	2,458,265	65	40	2
	Sandstone, cracking clays and shales	1,860,005	16	13	5
	Leached sands and siliceous sands	537,366	0	0	6
	Porous loamy soils, clay and friable earth	34,892	0	0	5
	Sandstones, hard acidic yellow and red soils	2,639,304	18	10	6
	Clays and loamy soils	803,627	2	3	6
	Shallow and stony leached loams	2,754	0	0	5
	Hard acidic mottled soils with leached sands	157,001	3	29	5
	Sandy or loamy red earths	106,873	0	0	5
	Moderate and shallow dark cracking clays	250,975	0	0	6
	Sandstones	1,589,702	1	0	6
	Shallow dark cracking clays	538,555	0	0	6
	Dark cracking clays	348,651	0	0	6
	Red and brown friable porous earth	1	0	0	5
	Rock outcrops and friable soils	83,011	0	0	5
	Loamy soils with clay	164,592	0	0	5
Geology	Phyllite and greywacke	1,835,300	42	7	5
	Sandstone, siltstone, shale and conglomerate	2,882,371	14	1	8
	Sand, silt, mud and gravel	929,694	7	2	7
	Granite, granodiorite, tonalite, diorite and gabbro	127,866	0	0	7
	Shale, conglomerate, sandstone, coal, siltstone, basalt and tuff	811,388	19	8	6
	Basaltic lavas with local rhyolite	927,150	0	0	8
	Andesitic to rhyolitic flows and volcaniclastic rocks	52,795	11	71	6
	Andesite	19,650	0	0	7
	Sandstone, mudstone and conglomerate	676,567	5	2	7
	Sandstone, siltstone, mudstone, coal and conglomerate	3,362,012	4	0	10
	Poorly lithified sandstone, conglomerate and mudstone	3,362,012	4	0	10
	Ferricrete and silcrete	263,124	3	3	7
	Basalt to gabbro plugs	192,005	1	1	7
LULC	Reservoir/dam	48,873	1	12	3
	Waste treatment and disposal	7,155	0	0	3
	Lake	9,314	0	0	3
	Marsh/wetland	978	0	0	3
	River	81,631	0	0	3
	Channel/aqueduct	779	0	0	3
	Nature conservation	767,825	0	0	4
	Managed resource protection	16,674	0	0	3
	Other minimal use	959,487	8	4	3
	Livestock grazing	6,937,978	24	2	6
	Production forestry	4,793	0	0	3
	Plantation forestry	105,294	0	0	3
	Grazing modified pastures	14,728	0	0	3
	Cropping	8,177	0	0	3
	Perennial horticulture	7,073	0	0	3
	Land in transition	226	0	0	3
	Irrigated modified pastures	177,342	0	0	3
	Irrigated cropping	420,877	3	4	3
	Irrigated perennial horticulture	11,025	0	0	3
	Irrigated seasonal horticulture	10,3824	0	0	3
	Intensive horticulture	1,397	0	0	3
	Intensive animal production	90,621	2	13	3
	Manufacturing and industrial	159,058	3	11	3
	Residential	1,873,566	27	8	3
	Services	471,135	35	43	2
	Utilities	13,453	0	0	3
	Transport and communication	21,731	0	0	3
	Mining	156,607	3	11	3
Distance from Roads(m)	0	401,007	8	26	9
	0–117.16	2,280,545	53	31	6
	117.16–351.48	1,932,712	20	13	9
	351.48–585.81	1,213,919	6	6	10
	585.81–937.29	1,343,246	6	6	10
	937.29–1405.93	1,314,392	7	7	10
	1405.93–1991.74	1,097,365	2	2	10
	1991.74–2811.87	1,031,440	2	2	10
	2811.87–4100.64	943,365	0	0	10
	4100.64–29876.13	913,630	2	2	10
Distance from Rivers (m)	0–489.65	1,241,245	31	31	7
	489.65–1305.74	1,482,252	49	42	6
	1305.74–2285.05	1,296,926	13	12	9
	2285.05–3590.81	1,313,062	4	3	10
	3590–5059.76	1,281,742	1	0	11
	5059.76–6691.94	1,234,808	0	0	11
	6691.94–9140.22	1,205,804	4	4	10
	9140.22–12894.24	1,142,553	4	4	10
	12894.24–18606.88	1,135,295	0	0	11
	18606.88–41620.6	1,137,934	0	0	11
Rainfall (mm/day)	1.86–2.81	1,245,402	3	2	10
	2.81–2.86	1,214,009	0	0	11
	2.86–2.92	1,278,634	9	8	10
	2.92–2.98	1,205,609	15	14	9
	2.98–3.04	1,459,866	13	10	9
	3.04–3.09	1,397,625	9	7	10
	3.09–3.17	1,344,633	6	5	10
	3.17–3.31	1,142,961	4	3	10
	3.31–3.42	1,049,373	18	19	9

The SPI range 157700.42–315400.84 had the highest flood susceptibility with a *Bel* value of 100. With regard to TWI, the highest flood potential was observed in the range 12.31–25.76 because this range showed the highest *Bel* (31) and lowest *Dis* (7) values. As the value of TWI increases, the water infiltration decreases, which can cause floods. Soil and geology also control water penetration and infiltration. The class of “Hard acidic yellow and red mottled soils” in soil and the class of “Andesitic to rhyolitic flows and volcaniclastic rocks” in geology received the highest *Bel* values of 40 and 71, respectively. The first three classes of distance from river, which were 0–489.65 m, 489.65–1305.74 m, and 1305.74–2285.05 m, received the highest *Bel* values. River proximity is one of the main factors in flood studies. The results showed that the areas closer to the river had higher chances of inundation. Heavy precipitation causes the ground to quickly become saturated and flood. This was confirmed by the acquired weight from EBF for the rainfall map. The highest rainfall class, 3.31–3.42, received the highest *Bel* value of 19 and a low *Dis* value of 9.

**Figure 4:**
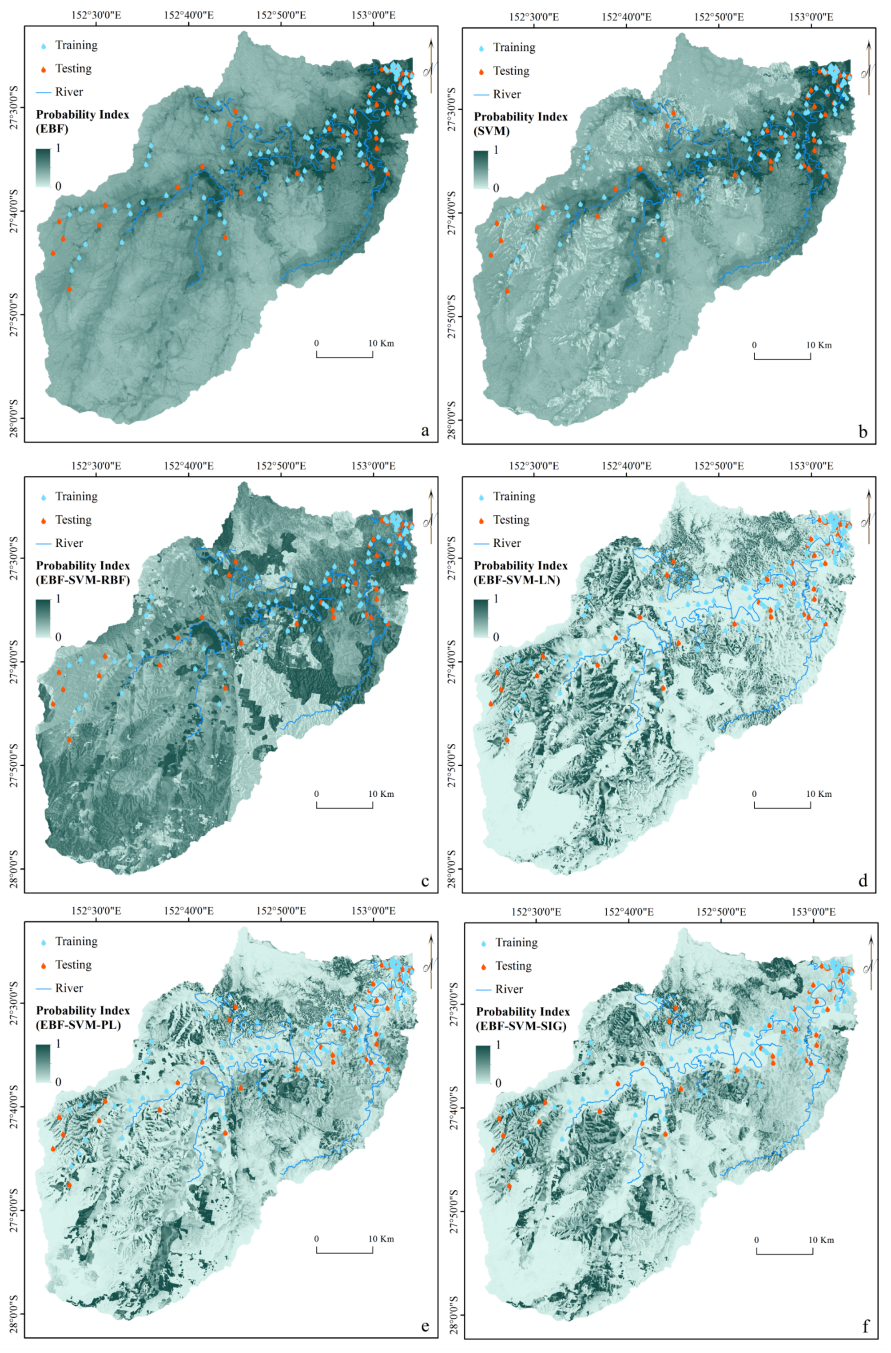
Flood probability index maps derived from: (A) individual EBF, (B) individual SVM, (C) ensemble EBF and SVM-RBF, (D) ensemble EBF and SVM-LN, (E) ensemble EBF and SVM-PL and (F) ensemble EBF and SVM-SIG.

As described in ‘Ensemble Modeling’, every conditioning factor was reclassified based on the derived EBF weights and used in SVM analysis to implement ensemble modeling. The ensemble method was applied using all four SVM kernels. The kernel parameters were derived from the cross-validation (Table 3). The final step involved the derivation of four flood probability indices. In addition, another flood probability map was generated using an individual SVM and an RBF kernel. The stand-alone SVM was undertaken using the original flood conditioning factors, which were not classified by the EBF results. Figure 4 illustrates the six flood probability index maps.

### Creations of flood susceptibility maps

**Figure 5:**
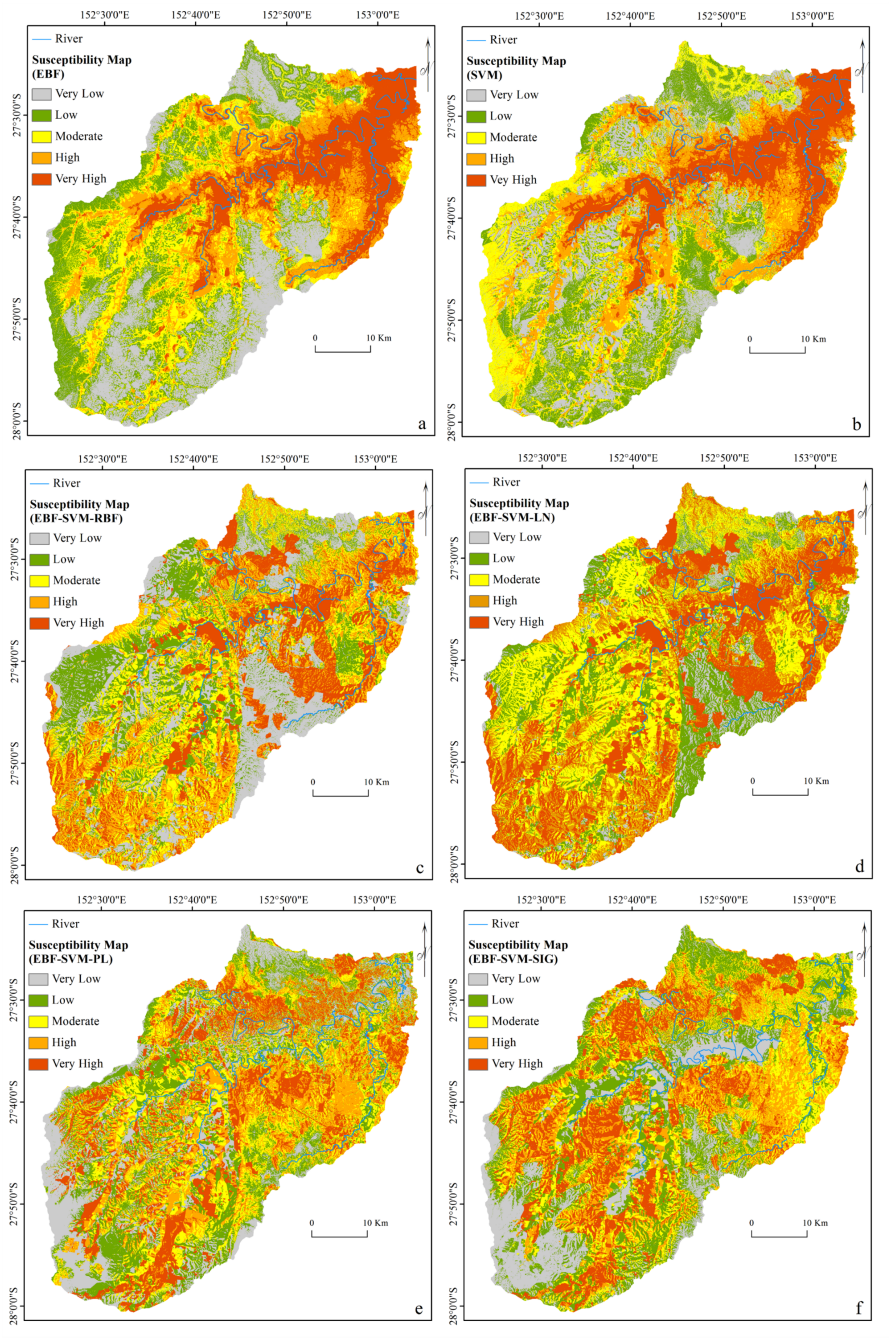
Flood susceptibility maps derived from: (A) individual EBF, (B) individual SVM, (C) ensemble EBF and SVM-RBF, (D) ensemble EBF and SVM-LN, (E) ensemble EBF and SVM-PL and (F) ensemble EBF and SVM-SIG.

To produce the flood susceptibility maps, the flood probability index has to be classified into different zones of susceptibility (Pradhan, 2013; Tehrany, Jones & Shabani, 2019). Natural break, equal interval, and quantile are some of the most commonly used methods in natural hazard probability index classification (Ayalew & Yamagishi, 2005). Two factors of data nature and data application influence the choice of classification method (Tehrany, Jones & Shabani, 2019). For instance, quantile is a technique that, without affecting the data, groups the pixels into same-size classes. This means that it groups equal numbers of pixels (area) into each susceptibility zone (Nampak, Pradhan & Manap, 2014). Therefore, it appears to be the most suitable method for classifying the flood probability index. To facilitate a reliable assessment of the impact of each class of a flood conditioning factor on flood occurrence, we attempted, where possible, to reduce the influence of the classification algorithm on the classes of the conditioning factor. However, natural break and equal interval might lead to a class with a large number of pixels and a class with few values (Chung & Fabbri, 2003). The flood susceptibility maps were produced by dividing each flood probability index into five susceptible classes of very low, low, moderate, high, and very high using a quantile method as seen in Fig. 5. The selected number of classes was based on the literature (Pham et al., 2017b; Termeh et al., 2018)

### Accuracy assessment

**Figure 6:**
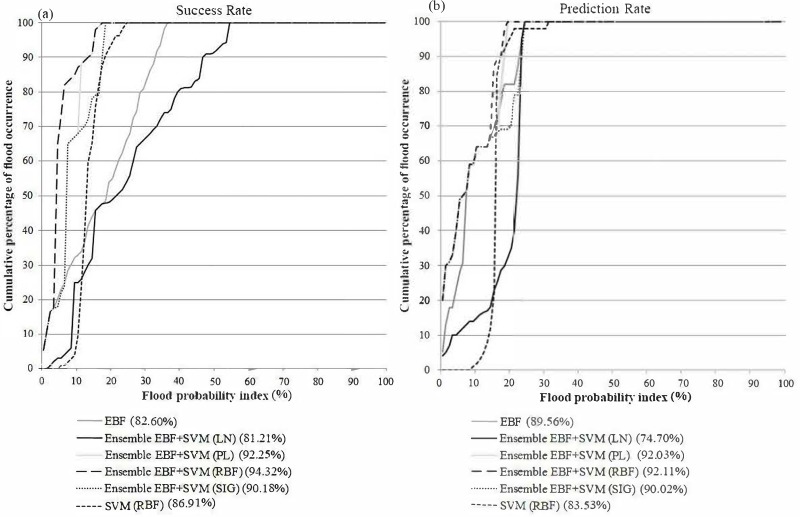
(A) Success rate, (B) prediction rate of flood susceptibility derived from (1) individual EBF, (2) individual SVM, (3) ensemble EBF and SVM-RBF, (4) ensemble EBF and SVM-LN, (5) ensemble EBF and SVM-PL and (6) ensemble EBF and SVM-SIG.

To evaluate the reliability of the derived susceptibility maps, an accuracy assessment was performed using the AUC method (Fig. 6). The AUC results showed that the highest prediction (92.11%) and success (94.32%) rates were achieved by the ensemble EBF-SVM–RBF method. The individual methods produced lower accuracies (EBF: 82.60% success rate and 89.56% prediction rate; SVM: 86.91% success rate and 83.53% prediction rate) compared to all the ensemble methods except the ensemble EBF-SVM–LN method (81.21% success rate and 74.70% prediction rate). The reason is that the linear kernel is not appropriate for use in non-linear phenomena such as flooding. Based on the achieved accuracies, the ensemble EBF and SVM method can be used instead of the individual methods to improve the accuracy of the final maps. This can help planners to recognize the most susceptible areas with higher certainty. Using the ensemble technique improved the success rate by 12% and 7% and the prediction rate by 3% and 9% over the individual EBF and SVM methods, respectively.

According to the results obtained in this study, ensemble modeling provided considerable advantages compared to the traditional methods. For example, the processing time for SVM was significantly reduced due to the pre-analysis of the flood conditioning factors. Hence, the factors were assessed and reclassified based on the EBF analysis and then used as an input for SVM. This quickened the machine learning process. In terms of cost, there are no direct differences among the methods in terms of performance; however, reducing the processing time in a large-scale analysis may speed up the management process, thereby reducing the damage costs in hazardous areas.

### Sensitivity analysis

As described earlier in the methodology section, every dataset includes an inevitable amount of uncertainty. The SA in this study was performed using the Jackknife test, and its outcomes are summarized in Table 5. The highest loss of performance or PRD ≈ 8.23 of the AUC method was achieved when slope was omitted from the conditioning factor dataset. This was followed by SPI (PRD ≈ 8.11) and geology (PRD ≈ 7.33). A higher PRD indicates that those conditioning factors provide specific information to the model that cannot be found in other factors. On the contrary, some of the conditioning factors did not represent strong contributions to the spatial prediction of flood occurrence such as distance from road (PRD ≈ 0.22), soil (PRD ≈ 0.65), and distance from river (PRD ≈ 0.71). These outcomes show that flood susceptibility mapping is highly sensitive to slope, SPI, geology, altitude, and LULC. Such an SA assists researchers in recognizing the most influential parameters in flood analysis. It is important to consider that these factors might be different in each study area.

**Table 5:** The Jackknife test results of variables when each conditioning factor is excluded in ensemble model.

Excluded factor	Decrease of AUC	Percent of relative decrease (PRD) of AUC
Slope	8.23	9.81
SPI	8.11	9.65
Geology	7.33	8.65
Altitude	6.98	8.20
LULC	6.16	7.17
Aspect	3.54	4.00
TWI	2.77	3.10
Curvature	0.87	0.95
Rainfall	0.73	0.80
Distance from river	0.71	0.78
Soil	0.65	0.71
Distance from road	0.22	0.24

## Conclusion

Proper and reliable techniques and strategies are required to assist governments and planners in identifying areas that are susceptible to floods and avoiding future urban development plans in these areas. Therefore, advancements in studies based on floods and available techniques are required to enhance our understanding the occurrence of floods varied climate and catchment conditions. To overcome the weaknesses of the stand-alone EBF and SVM methods, the more sophisticated ensemble methods can be used. In this study, a novel ensemble EBF-SVM method was developed, applied, and examined for the assessment of flood susceptibility mapping of the Brisbane Catchment, Australia, using GIS and SPSS Clementine V.14.2. Each of these methods is considered an efficient and powerful statistical technique. However, to enhance their performance, they were ensembled and used in this study. EBF and SVM were used to perform BSA and MSA, respectively. All four SVM kernels and their impacts were also considered. The ensemble method was applied four times using different kernels to identify the most proficient SVM kernel type. In addition, both EBF and SVM were used individually to obtain flood probability indices. The success rate and prediction rate of the AUC method were used to examine the strength and prediction capabilities of all the applied methods. The best accuracy was achieved by using the ensemble EBF-SVM–RBF method, with AUC of 94.32% and 92.11% for prediction and success rates, respectively. These values were approximately 6% higher than those obtained with the stand-alone models. The identified ensemble method offered the best fit for reasonable automatic flood conditioning parameter classification without any expert knowledge requirement. The performances of individual methods were enhanced by their integration. SVM offers different kernel types that can be selected based on the objective and data availability of each study. Each kernel is suitable for specific conditions, and each produces considerably different outcomes. Although the improvement in prediction was approximately 3% and 9% compared to the current individual EBF and SVM methods, respectively, the improvement is significant. Any increase in prediction accuracy can have a significant impact on flood mitigation planning, and the relevant method should be tested under different scenarios and implemented where possible.

##  Supplemental Information

10.7717/peerj.7653/supp-1 Supplemental Information 1 Spatial data GIS file

## Additional Information and Declarations

### Data Availability

The following information was supplied regarding data availability: The raw data are available as Supplemental File.
